# Associations between autistic and comorbid somatic problems of gastrointestinal disorders, food allergy, pain, and fatigue in adults

**DOI:** 10.1177/13623613241254619

**Published:** 2024-05-30

**Authors:** Yiran Li, Tian Xie, Harold Snieder, Catharina A Hartman

**Affiliations:** 1 Interdisciplinary Center Psychopathology and Emotion Regulation, Department of Psychiatry, University Medical Center Groningen, University of Groningen, Groningen, Netherlands; 2 Department of Epidemiology, University Medical Center Groningen, University of Groningen, Groningen, Netherlands

**Keywords:** autism, fatigue, food allergy, irritable bowel syndrome, pain

## Abstract

Autistic children often have comorbid somatic problems. However, in adulthood, this has been much less studied. We investigated the associations between autistic symptoms and irritable bowel syndrome, food allergy, pain, and fatigue in adulthood and examined sex and life-course differences herein. A total of 35,048 adults aged from 18 to 90 from the Lifelines Cohort Study reported autistic symptoms, irritable bowel syndrome, food allergy, pain, and fatigue. Associations between autistic and somatic problems were estimated by logistic and linear regression models. Higher autism scores were associated with more severe somatic problems with an odds ratio of 1.44 (95% confidence interval: (1.34, 1.55)) for irritable bowel syndrome, an odds ratio of 1.13 (95% confidence interval: (1.07, 1.20)) for food allergy, a regression coefficient (*b*) of 0.20 (95% confidence interval: (0.18, 0.22)) for pain and a regression coefficient (*b*) of 0.37 (95% confidence interval: (0.35, 0.39)) for fatigue. Associations were stronger in females than males for pain and fatigue. Associations declined with age for fatigue and food allergy and increased with age for irritable bowel syndrome and pain, in a subgroup with autism scores >98th percentile combined with symptom onset in childhood and high functional impairments. These findings are important for autistic adults and may aid in diagnosis, monitoring, and intervention.

**Lay Abstract:**

**What is already known about the topic?**

Autistic children frequently often have accompanying physical health problems. However, this has been much less studied in autistic men and women during adulthood.

**What does this article add?**

This is one of the first studies to investigate the associations between autistic and somatic problems in adults from the general population. Using a continuous measure of autistic symptom scores and a categorical definition of autism (referred to below as probable autism) which considered symptom severity, childhood age of onset, and functional impairment, we found that autistic problems and irritable bowel syndrome, food allergy, pain, and fatigue were associated in adults. Sex differences were present for pain and fatigue, for which the associations with autistic symptom scores were somewhat stronger in females than males. Regarding age differences, the associations with fatigue and having food allergy were more pronounced in younger adults. Conversely, older individuals had a higher risk of developing irritable bowel syndrome or experiencing pain if they met the criteria for probable autism.

**Implications for practice, research, or policy**

There is a need for providing routine programs of screening, assessment, and treatment of autism-related somatic problems and developing evidence-based interventions for autistic individuals. These could be tailored to the needs of specific autistic populations. For example, autistic females could be given extra attention about the potential presence of pain and fatigue, younger adults about the potential presence of food allergy and fatigue, and older adults concerning the potential presence of irritable bowel syndrome and pain.

## Introduction

Autism is a common mental health condition with a lifelong impact, with a prevalence of 1%–1.5% (Grove et al., 2019; Lyall et al., 2017). Somatic problems are rather common in autistic children, such as immune and gastrointestinal problems (Lefter et al., 2020), but information on the co-occurrence in adults is largely lacking in the literature (Cashin et al., 2018). The presence of comorbid somatic problems in autistic individuals is associated with reduced ability to work, overall decreased quality of life (Kuhlthau et al., 2018), and even increased mortality (Rydzewska et al., 2021; Sala et al., 2020). Studies focusing on somatic problems in autism across the lifespan of adults could be of major value for understanding disease etiology and improvement of diagnosis, management, and treatment of autism and somatic problems.

Research has shown that many comorbid somatic problems in autistic children are so-called functional somatic symptoms, which means that an organic cause to explain the somatic symptoms cannot (yet) be fully identified. For example, a recent study found that autistic-like features co-occur with functional somatic symptoms in adolescence. They found a stable, moderate association between autistic scores (measured by the Children’s Social Behavior Questionnaire) and an aggregated score of various functional somatic symptoms including gastrointestinal symptoms such as stomach pain and nausea together with pain and fatigue such as headache, overtiredness, and dizziness (Hogendoorn et al., 2023).

Among the various functional somatic symptoms, gastrointestinal symptoms are particularly highly comorbid with autism (Kostiukow et al., 2020; Lefter et al., 2020; Supekar et al., 2017). It has been reported that over half of autistic children have gastrointestinal comorbidities such as irritable bowel syndrome (IBS) (Penzol et al., 2019; Sanctuary et al., 2019). IBS is characterized by a group of symptoms including abdominal pain, diarrhea, and constipation (Grundmann & Yoon, 2010). It is one of the most common functional gastrointestinal disorders and one of the most frequent complications in the autistic population (Grundmann & Yoon, 2010). A retrospective review of electronic medical records from local hospitals in the United States of autistic children and young adults under age 35 years (*N* = 14,381) showed that the prevalence of bowel disorders in autistic individuals was more than twofold higher than in the general patient population (*N* = 2,393,778) (11.7% vs 4.5%) (Kohane et al., 2012).

Food allergy, which is related to gastrointestinal symptoms (Rajani et al., 2020; Wasilewska & Klukowski, 2015), is also associated with autism. A meta-analysis study reported that the odds ratio (OR) for autistic individuals (children and young adults) with food allergy was 2.22 compared with the non-autistic group, confirming a strong relationship between food allergy and autism (Wang et al., 2021). However, most of these studies have so far been conducted among autistic children, and the knowledge available for adults is very limited.

In addition to gastrointestinal problems and food allergy, autistic individuals often have altered sensitivity to sensory stimuli, which may affect somatic symptom perception, such as pain (Sala et al., 2020). However, associations of autism with pain have been rarely studied and the available evidence is inconclusive. One previous meta-analysis study investigated gastrointestinal symptoms including abdominal pain in autistic children, ranging from birth to 18 years old. The study included 15 studies where autism was categorically defined mostly by rating scales, chart reviews, or community provider using diagnostic criteria. Gastrointestinal symptoms were assessed mainly through caregiver reports (73%) or medical chart reviews (27%). The findings showed a higher rate of abdominal pain in autistic children (OR = 2.45) across these varied studies (McElhanon et al., 2014). Regarding general pain, only one cross-sectional study explored the association in children and adolescents with chronic debilitating pain (*N* = 146). The results showed no difference in experienced pain intensity, pain duration, and pain frequency between children with and without clinically significant autistic traits (Lipsker et al., 2018).

Similarly, autistic individuals may also have a higher risk of experiencing fatigue due to an overload of sensory and social interactions (Keville et al., 2021). However, research studying the relationship is limited, with only one study reporting no significant associations. The study compared autistic symptom scores of adults with chronic fatigue syndrome (*N* = 59) and a non-autistic comparison group (*N* = 53). The authors did not find a difference in autistic symptoms between the two groups (Bileviciute-Ljungar et al., 2018). Notably, the available studies examining the association of general pain and fatigue had small sample sizes, which may have resulted in limited statistical power to identify any potential association (Bileviciute-Ljungar et al., 2018; Lipsker et al., 2018).

Previous studies illustrated that autism and somatic symptoms often co-occur with the strongest evidence on gastrointestinal symptoms and food allergy, and some (partly conflicting) evidence on comorbid pain and fatigue. However, some gaps remain in the existing research. First, studies in adulthood on comorbid somatic symptoms including IBS, food allergy, pain, and fatigue are quite rare. Second, most of the existing studies examined the relationships in persons with categorically defined autism (Asztély et al., 2019; Holingue et al., 2018; Lefter et al., 2020; McElhanon et al., 2014; Wang et al., 2021). However, autism is generally not seen as a discrete condition; individuals exhibit autistic symptoms with a wide range, and we may lose important information as well as statistical power if studies are exclusively focused on the extreme and most severe end of this continuous distribution (Ruzich et al., 2015).

Third, only a few studies explored potential age and sex differences in co-occurrence patterns. Some somatic symptoms, such as widespread musculoskeletal pain and fatigue, have a peak onset in adolescence or adulthood (Williams & Gotham, 2022). Therefore, somatic symptom burden may be more pronounced in autistic adults than in children. Similarly, some somatic problems are more prevalent in females (e.g. IBS, pain, and fatigue) in the general population and it is important to study if this likewise holds for autism, which is more frequent in males (Faro et al., 2016; Kim & Kim, 2018; Sorge & Totsch, 2017; Werling & Geschwind, 2013).

In sum, individual differences in somatic symptom burden in autistic men and women across the lifespan remain poorly documented; it is important to expand research beyond childhood and investigate the relationship with sex and age in adults. A comprehensive understanding of such age- and sex-specific associations is important for stratifying risk and providing individualized treatment recommendations in autistic individuals. In the present study, we investigated the associations between autistic and somatic problems, including IBS, food allergy, musculoskeletal pain, and fatigue in a large sample (*N* = 35,048) from the general population. Our primary objective was to examine these associations using a (multidimensional) continuous measure of autism. In addition, our secondary objective was to examine the associations using a categorical measure of autism to align with existing literature.

## Method

### Participants

**Figure 1.**
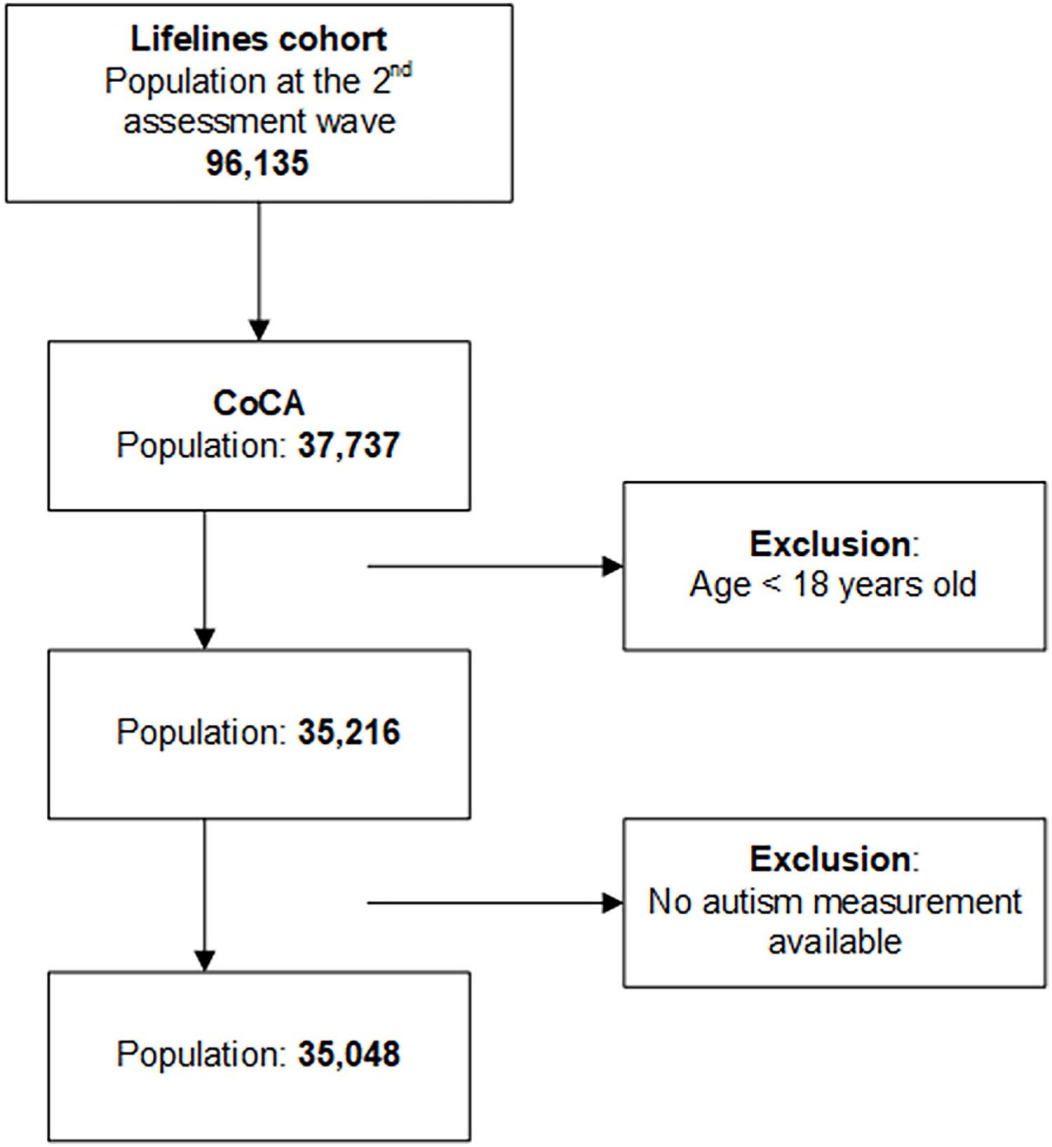
Flowchart of the inclusion of the study population.

The study was embedded in the Lifelines Cohort Study. Lifelines is a multidisciplinary prospective population-based cohort study examining in a unique three-generation design the health and health-related behaviors of 167,729 persons living in the north of the Netherlands (Scholtens et al., 2015; Sijtsma et al., 2021). It employs a broad range of investigative procedures in assessing the biomedical, sociodemographic, behavioral, physical, and psychological factors which contribute to the health and disease of the general population, with a special focus on multimorbidity and complex genetics. The participants were recruited between 2006 and 2013 at baseline and followed up from 2014 onwards. The second wave of assessment has been performed between 2014 and 2019. In Lifelines, an add-on study was conducted from 2017 to 2019 as a part of the European Union (EU)-funded Comorbid Conditions of Attention-Deficit/Hyperactivity Disorder (ADHD) (CoCA) consortium research. The add-on study included 35,216 adults. The participants filled in a digital survey that assessed the presence, age of onset, and impairment of various psychiatric problems such as autism. The Lifelines Cohort Study and the CoCA add-on study were approved by the ethics committee of the University Medical Centre Groningen and all participants signed an informed consent form (Stolk et al., 2008). For the current study, we used cross-sectional data, collected at the second assessment wave, of 35,048 adults aged from 18 to 90 years who had available measurements of autism (Figure 1).

### Measurements

#### Autism

Autism was measured by the 44-item Adult Social Behavior Questionnaire (ASBQ) (Horwitz et al., 2016, 2020). This is a validated questionnaire to quantify autism symptoms in adults. Items are scored on a 3-point Likert-type scale ranging from *does not apply* (0) to *clearly applies to you* (2) and result in a sum score ranging from 0 to 88 (Horwitz et al., 2016, 2020). The ASBQ measures autistic problems in six subscales: reduced contact, reduced social insight, reduced empathy, violation of social conventions, difficulty with change, and stereotyped sensory-motor behaviors. A continuous total score was used in the main analyses, with higher scores indicating more severe autism symptoms. This score was standardized into a *Z*-score distribution to facilitate interpretation. In secondary analyses, we categorized the continuous distribution. We used one proxy for the possible presence of clinical autism, allowing to evaluate whether conclusions would be different when focused on the extreme and impaired part of the autism continuum. This was accomplished by using a cut-off score for autism score of 2% in our sample. In addition, it was required that the age of onset was before 18 years old and an impairment level of at least 5 points on a scale from 1 to 10 was reported. Together, these decision rules created a group of 1.2% of the study sample identified with probable autism. Thus, we probed a group which approximates the current estimate of the prevalence of autism in the general population (Brugha et al., 2011; Lyall et al., 2017). Finally, additional secondary analyses were performed on the six subscales of the ASBQ using standardized *Z*-scores. To understand whether specific groups of autistic symptoms are associated with specific types of somatic symptoms, exploratory analyses were conducted to examine associations between the subscales of the ASBQ and somatic symptoms (e.g. the association between stereotyped motor behaviors and pain).

#### Somatic problems

##### IBS

IBS was assessed by the Rome III IBS Diagnostic Questionnaire, which is a validated and reliable survey to assess whether individuals meet the diagnostic criteria for IBS according to Rome III criteria (Mostafa, 2008). The questionnaire contains 27 items assessing the occurrence, frequency, and duration of bowel complaints (e.g. abdominal pain, bowel movement) and other related symptoms (e.g. change in form or appearance of stool). Participants were identified as “having IBS” if they met the following diagnostic criteria: (1) having recurrent abdominal pain or discomfort at least 1 day per week; (2) symptom duration of at least 6 months; (3) having additional symptoms (at least 2 of the following 3 symptoms should be indicated): a. Improvement with defecation; b. onset associated with a change in frequency of stool; c. onset associated with a change in form or appearance of stool; (4) for women, abdominal pain or discomfort should not only occur during menstrual bleeding. Other participants were classified as “not having IBS.” The instrument has shown good specificity and sensitivity compared with diagnoses made by experienced clinicians (Dorn et al., 2009; Whitehead & Drossman, 2010).

##### Food allergy

Food allergy was measured by collecting information on 60 foods that may cause an allergic response (e.g. apple, peanut, egg, milk), related symptoms (e.g. diarrhea, urticaria, wheezing) consistent with the immediate allergic reactions to foods, and other characteristics of food allergy including (1) Who diagnosed the food allergy? (2) Do you have an adrenalin auto-injector/Epipen/Anapen/Jext? (3) Were you tested in a 2-day double-blind oral food challenge? (4) Did this test show that you are allergic to at least one food? (5) Which food item triggers the most severe allergic reaction? (6) How quickly do these symptoms appear? (7) Which amount causes these symptoms? (8) How long do these symptoms persist? (Westerlaken-van Ginkel et al., 2020). Participants were classified into “likely having a food allergy,” “indeterminate,” and “not having a food allergy.” Subjects were classified as “likely having a food allergy” if they reported (1) at least one food that yielded an immediate allergic reaction to food; (2) at least one symptom consistent with the immediate allergic reactions to food; and (3) other characteristics of food allergy consistent with immediate allergic reactions to food. “Indeterminate” subjects were those who reported food allergy according to the following criteria: (1) only symptoms to foods uncommon or unproven to be elicitors of immediate allergic reactions; OR (2) only symptoms other than those consistent with immediate allergic reactions to foods; OR (3) only symptoms and/or foods associated with other disorders (such as lactose intolerance); OR (4) one or more other characteristics which are not consistent with allergic reactions to food. Subjects were classified as having “no food allergy” if none of the above criteria were applied.

##### Pain

Pain was measured by the Widespread Pain Index (WPI). The WPI is a widely used 19-point checklist that assesses the presence of musculoskeletal pain or tenderness in 19 specific body regions in the past 1 week (e.g. shoulder, hip, arm, etc.), with each affected region scoring one point (Wolfe et al., 2010, 2011). Item mean score was calculated and used for the analyses, with higher scores indicating more widespread musculoskeletal pain. The score was standardized into a *Z*-score distribution to facilitate interpretation.

##### Fatigue

Fatigue was assessed by the Checklist Individual Strength, a 20-item fatigue questionnaire (Vercoulen et al., 1999). It measures fatigue-related problems in four dimensions in the past 2 weeks, including subjective fatigue, concentration, motivation, and physical activity (Vercoulen et al., 1994). A 7-point Likert-type scoring scheme is used for each item. Item mean score was calculated and used for the analyses, with higher scores indicating higher levels of fatigue. The score was standardized into a *Z*-score distribution to facilitate interpretation.

#### Covariates

We adjusted for age, sex, education years, employment, income, and neighborhood socioeconomic status (NSES) in the analyses, which were covariates potentially associated with autism symptom severity and somatic symptoms (Halladay et al., 2015; Rai et al., 2012; Williams & Gotham, 2022). Education years were the years of schooling. For employment, measurement of the Standard International Occupational Prestige Scale 2008 (SIOPS08) was used (Hoveling et al., 2021). SIOPS08 is a continuous scale ranging from 0 to 100, with higher scores indicating higher occupational prestige (Ganzeboom & Treiman, 1996). Income was calculated using the net income per month per family divided by the number of people living on the income. NSES was determined according to the status score derived from the Dutch governmental institute for statistical analyses regarding societal issues (CBS Statistics Netherlands) and the Netherlands Institute for Social Research (den Ridder et al., 2020). The status score is a measure of the social status of a neighborhood at the level of a 4-digit postal code in the Netherlands. It consists of the inhabitants’ average educational level, job prospects, and income. NSES score is *mean*-centered and ranges from approximately −8 to +3, with a higher score indicating a better social status. This measure was linked to the Lifelines data.

### Statistical analyses

Logistic and linear regression models were employed to estimate the associations between autism and, respectively, categorical (IBS and food allergy), and continuous outcomes (pain and fatigue). Since food allergy in the study was an ordinal variable, ordinal logistic regression was used to estimate the association. Continuous variables (autism, pain, fatigue, and covariates age, education years, employment, income, and NSES) were standardized to *Z-*scores to facilitate interpretation. Standardized age and age^2^ were included as independent variables in the models to allow the nonlinear relationship between age and somatic outcomes. Interactions between autism and sex, autism and age, and autism and age^2^ were also included in the models. Data cleaning and statistical analyses were performed using R Statistical Software version 4.1.3 and package “mass” was used for logistic regression analyses (v4.1.3; R Core Team, 2023; Ripley et al., 2013). A two-sided *p* < 0.05 was considered statistically significant. In secondary analyses, we reran the models by using the categorical autism. Also, we reran the models with the six autism subscales as predictors, respectively.

### Community involvement

There was no community involvement in the current study.

## Results

Characteristics of the total sample and for men and women separately are shown in Table 1. The *mean* (*SD*) age of the participants was 49.5 (12.5) years and 41.8% (14653/35048) were men. Men had higher autism score compared with women (12.7 vs 9.6). IBS was present in 4.4% (1547/35048) of the sample and 7.6% (2648/35048) had a “likely food allergy.” Women had a higher prevalence of IBS, food allergy, and higher scores of pain and fatigue compared with men (for IBS: 5.7% vs 2.6%; for “likely food allergy”: 9.5% vs 4.9%; for pain: 0.15 vs 0.10; for fatigue: 2.70 vs 2.51).

**Table 1.** Characteristics of the study sample.

	Total (*N* = 35,048)	Female (*N* = 20,395)	Male (*N* = 14,653)
Age, *mean* (*SD*)	49.5 (12.5)	48.3 (12.2)	51.1 (12.6)
Education years, *mean* (*SD*)	14.9 (4.2)	14.8 (4.1)	15.1 (4.2)
Employment prestige, *mean* (*SD*)	45.5 (13.3)	44.3 (13.5)	47.2 (12.7)
Income, *mean* (*SD*)	1710 (529)	1670 (533)	1770 (519)
NSES, *mean* (*SD*)	−0.55 (1.08)	−0.56 (1.08)	−0.54 (1.09)
Autism, *mean* (*SD*)	10.9 (11.0)	9.6 (10.3)	12.7 (11.7)
Probable autism^a^, *n* (%)	433 (1.2)	198 (1.0)	235 (1.6)
IBS, *n* (%)	1547 (4.4)	1165 (5.7)	382 (2.6)
Food allergy, *n* (%)			
Likely food allergy	2648 (7.6)	1930 (9.5)	718 (4.9)
Indeterminate	1101 (3.1)	779 (3.8)	322 (2.2)
No food allergy	31,299 (89.3)	17,686 (86.7)	13,613 (92.9)
Pain, *mean* (*SD*)	0.13 (0.14)	0.15 (0.15)	0.10 (0.12)
Fatigue, *mean* (*SD*)	2.62 (1.17)	2.70 (1.19)	2.51 (1.11)

NSES: neighborhood socioeconomic status; IBS: irritable bowel syndrome.

a Probable autism was classified by the following criteria: first, the total score exceeded the cut-off score according to the prevalence in the general population which was set to 2%. Second, the age of onset was before 18 years old and an impairment level of at least 5 points on a scale from 1 to 10 was reported.

**Figure 2.**
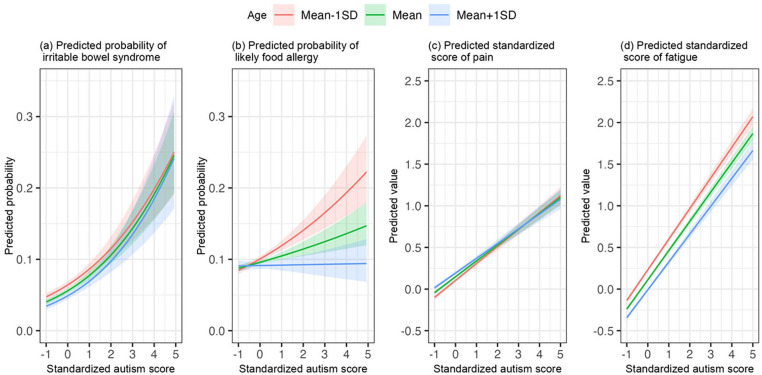
Associations between autism and somatic problems by age. *SD*: standard deviation. Logistic regression models were used for irritable bowel syndrome and likely food allergy. Linear regression models were used for pain and fatigue. Figure 2(b) to (d) show additive and interactive effects of age and autism score. The associations of autism with food allergy and fatigue were stronger in younger adults. Pain score was higher in older than younger adults if they had low autism scores, while in adults with high autism scores, the association with pain did not depend on age. Figure 2(a) shows the additive effects of age and autism score.

**Figure 3.**
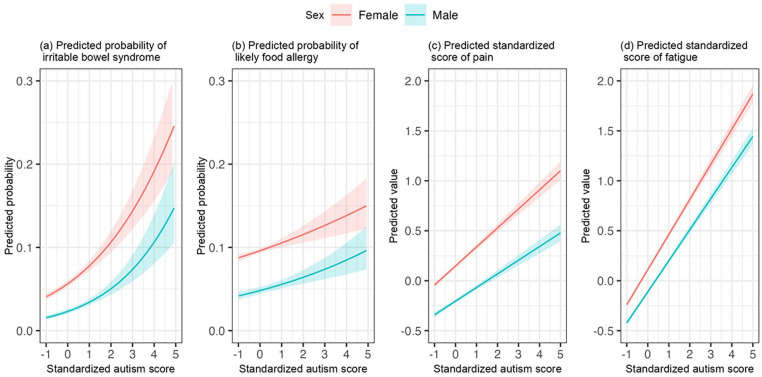
Associations between autism and somatic problems by sex. Logistic regression models were used for irritable bowel syndrome and likely food allergy. Linear regression models were used for pain and fatigue. Figure 3(c) and (d) show additive and interactive effects of sex and autism score. The associations of autism with pain and fatigue were stronger in females. Figure 3(a) and (b) show additive effects of sex and autism score.

Table 2 shows the results of the regression analyses examining the associations between autistic symptom scores and somatic problems adjusting for all covariates. Overall, effect of autistic symptom scores on somatic problems were positive, indicating that higher autistic symptom scores were associated with more somatic problems. Specifically, each unit (equal to 1 *SD*) increase in autistic symptom score was associated with 1.44- and 1.13-times higher odds of having IBS and food allergy, respectively (for IBS: OR = 1.44, 95% CI: (1.34, 1.55); for food allergy, OR = 1.13, 95% CI: (1.07, 1.20)). Similarly, each unit (equal to 1 *SD*) increase in autistic symptom score was associated with 0.20 and 0.37 units increase in pain and fatigue, respectively (for pain, *b* = 0.20, 95% CI: (0.18, 0.22); for fatigue, *b* = 0.37, 95% CI: (0.35, 0.39)). The regression analyses further indicate negative effects of age on somatic problems, indicating that, overall, younger age was associated with more severe somatic problems. (for IBS: OR = 0.87, 95% CI: (0.81, 0.93); for food allergy, OR = 0.95, 95% CI: (0.91, 0.99); for pain, *b* = −0.03, 95% CI: (−0.04, −0.02); for fatigue, *b* = −0.12, 95% CI: (−0.13, −0.11)). The associations between autistic symptoms and somatic problems depended on the participant’s age and sex, as indicated by significant interaction effects (Figures 2(b) to (d) and 3(c) and (d)). For food allergy, the association with autistic symptoms was somewhat stronger in younger than in older adults (OR _autism × age_ = 0.91, 95% CI: (0.88, 0.95), Figure 2(b)). Likewise for fatigue, the association with autistic symptoms was somewhat stronger in younger than in older adults (*b*_autism × age_ = −0.02, 95% CI: (−0.03, −0.01), Figure 2(d)). Also, an age effect was found in the association of autistic symptoms with pain (*b*_autism × age_ = −0.01, 95% CI: (−0.03, −0.001), Figure 2(c)). Figure 2(c) shows that in case of low autistic symptom scores, pain was higher in older than younger adults; however, in case of high autistic symptom scores, pain was high for all ages. With regard to sex effects, the associations of autistic symptoms with pain and fatigue were stronger in females (pain, *b*_autism × sex (male)_ = −0.05, 95% CI: (−0.08, −0.03), Figure 3(c); fatigue: *b*_autism × sex (male)_ = −0.04, 95% CI: (−0.06, −0.02), Figure 3(d)). No significant age or sex effects were found in the association between autistic symptoms and IBS.

**Table 2.** Associations between autism score and somatic problems.

Predictors	Somatic problems			
	Categorical outcomes		Continuous outcomes	
	Irritable bowel syndrome (yes/no)	Food allergy (likely food allergy/indeterminate/no)	Pain (scale from −0.91 to 6.15)	Fatigue (scale from −1.39 to 3.75)
	OR	OR	*b*	*b*
	(95% CI)	(95% CI)	(95% CI)	(95% CI)
Autism score^a^	1.44***	1.13***	0.20***	0.37***
	(1.34, 1.55)	(1.07, 1.20)	(0.18, 0.22)	(0.35, 0.39)
Sex (male)^b^	0.40***	0.48***	−0.35***	−0.22***
	(0.34, 0.46)	(0.44, 0.52)	(−0.38, −0.33)	(−0.25, −0.20)
Age	0.87***	0.95*	−0.03***	−0.12***
	(0.81, 0.93)	(0.91, 0.99)	(−0.04, −0.02)	(−0.13, −0.11)
Age^2^	0.99	0.94***	0.04***	−0.03***
	(0.94, 1.03)	(0.91, 0.97)	(0.03, 0.06)	(−0.04, −0.02)
Education years	1.00	1.06*	−0.05***	−0.01
	(0.93, 1.07)	(1.01, 1.11)	(−0.06, −0.03)	(−0.02, 0.01)
Employment	1.01	1.03	−0.01	0.01
	(0.94, 1.08)	(0.98, 1.07)	(−0.01, 0.01)	(−0.01, 0.02)
Income	0.95	1.04	−0.04***	−0.04***
	(0.89, 1.01)	(1.00, 1.08)	(−0.06, −0.03)	(−0.06, −0.03)
NSES	0.95	0.98	−0.01	−0.01
	(0.90, 1.00)	(0.95, 1.02)	(−0.02, 0.01)	(−0.02, 0.01)
Autism × sex (male)^b^	1.06	1.05	−0.05***	−0.04***
	(0.95, 1.18)	(0.97, 1.13)	(−0.08, −0.03)	(−0.06, −0.02)
Autism × age	1.02	0.91***	−0.01**	−0.02**
	(0.97, 1.08)	(0.88, 0.95)	(−0.03, −0.001)	(−0.03, −0.01)
Autism × age^2^	0.98	0.97	−0.01**	−0.02***
	(0.94, 1.02)	(0.94, 1.00)	(−0.02, −0.003)	(−0.03, −0.01)

OR, odds ratio; CI: confidence interval; NSES: neighborhood socioeconomic status; *b*: coefficient.

a Autism score was standardized and the scale was from −0.99 to 5.84.

b Female was the reference category.

* *p* < 0.05, **p < 0.01, ***p < 0.001.

We found similar associations based on the dichotomous outcome measure of “probable autism” in relation to IBS, pain, and fatigue (Supplementary Table S1, Figures S1 and S2). Thus, participants with a categorically defined probable autism had more somatic problems including IBS, pain, and fatigue. The association between categorical probable autism and food allergy was not significant as based on the upper and more severely impaired 1.2% of the distribution. However, estimates (Table 2) and the plot (Figure 2(b)) in the main analyses were highly similar to the findings from this secondary analysis using categorical probable autism, indicating that this non-significant finding resulted from lower statistical power using categorical probable autism. Furthermore, we identified an interaction effect with age indicating a stronger association of categorical probable autism with IBS and pain in older adults (Supplementary Table S1, Supplementary Figures S1a and S1c). These were not found in our main analyses, which revealed a stable association across the lifespan for individuals with high autistic symptom scores (Table 2, Figure 2(a) and (c)). Like in the main analyses, a significant interaction effect between probable autism and sex for pain was identified indicating a stronger effect in females (Supplementary Table S1, Supplementary Figure S2c). The interaction of probable autism with sex was not found for fatigue; however, estimates in the main analyses (Table 2) and the plot (Figure 3(d)) were highly similar to the corresponding findings in secondary analyses, indicating that the non-significant interaction was due to reduced statistical power.

Our secondary analyses of the scores on the six ASBQ subscales separately showed that the results across all subscales were highly consistent with the main results (Supplementary Table S2).

## Discussion

This is one of the first efforts to investigate the associations between autistic and somatic problems in adults from the general population. We found that autistic symptoms and IBS, food allergy, pain, and fatigue were associated. Associations for pain and fatigue depended somewhat on the participant’s sex and were stronger for females. Associations were stronger for younger than older adults for fatigue and food allergy, and were stronger for older than younger adults, in the subgroup with high functional impairments, for IBS and pain.

The associations of autistic symptoms with IBS and food allergy were consistent with most of the previous studies which were predominantly performed in children (Kostiukow et al., 2020; Lefter et al., 2020; Wang et al., 2021), and one small-sized study which included young adults who were under 35 years old (Kohane et al., 2012). Our study added to this by confirming the association in the adult general population, based on both multidimensional continuous and dichotomous definitions of autism. This association was not restricted to children and was not only present in referred, and presumably more severely affected adults, but also in adults identified in the general population (regardless of referral status) which were on average less severely affected. In addition, the associations held for all aspects of autistic symptoms. In the current study, the association of autistic symptoms with food allergy was not identified in the secondary analyses that focused on the most severely affected impaired 1.2% of the distribution. But as noted in the “Results” section, given highly similar estimates and plots, we attributed this to the reduced statistical power using categorically defined autism.

We additionally found that autism symptoms in adults were related to pain and fatigue, which have so far been much less studied among both children and adults compared with IBS and food allergy. One study suggested that the large majority of women (76.6%) who were diagnosed with autism had chronic pain (Asztély et al., 2019). However, other studies reported no associations with pain or fatigue (Bileviciute-Ljungar et al., 2018; Lipsker et al., 2018). These studies were limited by small sample sizes (less than 200 participants), which may partly explain the heterogeneity in findings. Our study thus added to the existing knowledge based on the associations of autistic symptoms with pain and fatigue by showing robust associations both when analyzing the autism continuum including all its dimensions, as well as when focusing on the most severely affected individuals.

It has rarely been investigated if there are age and sex differences in the presence of somatic problems in autism. One study suggested that among children with chronic pain, girls were more likely to have co-occurrent autism (Lipsker et al., 2018). Interpretation of the findings was somewhat limited due to the failure to adjust for potential confounders and the small sample size (*N* = 102 girls; total sample *N* = 146) with autism (Lipsker et al., 2018). In general, the lower prevalence rate of autism in women makes it difficult to recruit adequate numbers of female participants with autism and to examine sex-specific comorbidity patterns (Werling & Geschwind, 2013). Note that also in our present large study, when based on the categorical outcome measure “probable autism,” we had a predominantly male sample, illustrating the difficulty to establish sound knowledge on female adults with autism (Werling & Geschwind, 2013). When studying the full autism continuum, we found that with increasing autistic symptom scores, women were more vulnerable than men to suffer from pain and fatigue; this, on top of the overall higher scores of women on pain and fatigue irrespective of autistic symptom scores (Faro et al., 2016; Sorge & Totsch, 2017), which was also shown in our results. This likewise held in the more impaired upper 1.2% of the distribution; although the effect was not significant for fatigue in these secondary analyses, which, as already noted in the results, based on highly similar estimates and plots, we attributed to lower statistical power.

Although autism is a lifelong neurodevelopmental condition, and there is an obvious need for basic knowledge of lifespan changes, few studies have focused on age-specific patterns of co-occurrence. Here, when studying the full autism continuum, we found that younger people were more susceptible than older people to have food allergy and fatigue with increasing autistic symptom scores. Previous studies suggested that the prevalence of food allergy and fatigue decreases with age in the general population (Akerstedt et al., 2018; Engberg et al., 2017; Gupta et al., 2019; Sicherer et al., 2020; Westerlaken-van Ginkel et al., 2020), which is confirmed by our results. We added to the literature that the association with autistic symptoms also decreased with age, which was most outspoken for the association of autistic symptoms with food allergy to the extent that it was absent in older-aged adults (Figure 2(b), S1b). This was not the case for fatigue in which age reductions in association with autistic symptoms were much more subtle. For pain and IBS, our main analyses indicated that the association with pain and IBS held similarly across the full lifespan. However, in the impaired, upper 1.2% of the distribution, we found that the associations were stronger in older compared with younger adults. This is likely due to autism severity: in addition to their high scores, individuals in the upper 1.2% were required to report an early age of onset and substantial impairments in daily life. This resulted in a subsample that was different in that they were more often male (male prevalence was 54.3% compared with 41.8% in the total sample). Regarding IBS, our results in the upper 1.2% distribution were in line with findings from a previous study which suggested that the prevalence of bowel disorders was higher in the individuals with autism who were older than 35 years old compared with the 18–35 age group with autism (Supekar et al., 2017). Notably, this is quite distinct from the prevalence in the general population, which has a declining trend of IBS prevalence with increasing age (in our sample (Table 2 and Figure 2(a)), as well as in Canavan et al., 2014 and Chatila et al., 2017)).

The associations found in the current study indicate emerging hypotheses involving the “gut–immune–brain axis,” shared genetic variants, and altered sensitivity to sensory stimuli in autistic individuals (Azhari et al., 2019; Grove et al., 2019; Keville et al., 2021). Previous studies found abnormal gut-derived metabolite patterns that are strongly associated with gastrointestinal symptoms and food allergy among autistic children (Dan et al., 2020; Mortera et al., 2022; Needham et al., 2021; Rachid & Chatila, 2016). Furthermore, as we mentioned before, pain and fatigue may be amplified as a protection mechanism in response to intensified stimuli that overload the sensory systems in autistic individuals (Hoffman et al., 2023). Alternatively, studies investigating genetic contributions suggested that autism has a shared genetic basis with (part of) the currently studied somatic problems. For example, a genetic association between autism and tiredness has been identified (Grove et al., 2019). Another recent study found that polygenic risk scores of autism were associated with comorbid allergy and low pain tolerance, likewise suggesting shared genetic risk variants (Klein et al., 2022).

### Strengths

First, this study was embedded in a large, well-characterized, population-based cohort, which is broadly representative of the general Dutch population (Klijs et al., 2015). We included adults of all ages across the full range of autistic symptoms to examine associations in the general population. Second, autism was included both as multidimensional continuous and categorical variables, adding evidence to the overall patterns of co-occurrence and providing the link to the clinical literature using, for example, case–control studies. Third, we characterized autistic problems across sex and age, providing potential clues for the improved understanding of the somatic problems and the development of sex- and age-specific interventions.

### Limitations

There are several limitations in this study. First, both autistic and somatic problems were ascertained based on self-reported information, rather than by clinicians, which may lead to potential self-report bias. However, the validated scales used in the study have shown high specificity and sensitivity, which may limit the bias. Second, temporal relationships or causal inference could not be established in our current cross-sectional study.

## Conclusion and implications

Autistic symptoms and IBS, food allergy, pain, and fatigue co-occur. This was previously shown particularly in childhood (Kostiukow et al., 2020; Lefter et al., 2020; Wang et al., 2021) and currently in adulthood, in line with previous studies with small sample sizes (Kohane et al., 2012; Lipsker et al., 2018). Sex differences were present for pain and fatigue, for which the association with autistic symptoms was somewhat stronger in females. Age-declining associations with autistic symptoms were found for fatigue and particularly food allergy, and age-increasing associations with autistic symptoms for IBS and pain in adults who were most severely impaired. Interventions and treatment focusing on somatic symptoms could be tailored to the needs of vulnerable populations with autism symptoms. For example, females could be given extra attention with regard to the potential presence of pain and fatigue, younger adults with regard to the potential presence of food allergy and fatigue, and older adults with regard to the potential presence of IBS and pain. However, these interaction effects were more subtle than overall associations, indicating the relevance of IBS, food allergy, pain, and fatigue for all autistic individuals. If unnoticed or neglected, these risks could result in long-term detrimental impacts on health and quality of life over the lifespan. Therefore, there is a need for providing routine programs of screening, assessment, and treatment of autism-related somatic problems, and developing evidence-based interventions for autistic individuals.

## Supplemental Material

sj-docx-1-aut-10.1177_13623613241254619 – Supplemental material for Associations between autistic and comorbid somatic problems of gastrointestinal disorders, food allergy, pain, and fatigue in adults Supplemental material, sj-docx-1-aut-10.1177_13623613241254619 for Associations between autistic and comorbid somatic problems of gastrointestinal disorders, food allergy, pain, and fatigue in adults by Yiran Li, Tian Xie, Harold Snieder and Catharina A Hartman in Autism

sj-docx-2-aut-10.1177_13623613241254619 – Supplemental material for Associations between autistic and comorbid somatic problems of gastrointestinal disorders, food allergy, pain, and fatigue in adults Supplemental material, sj-docx-2-aut-10.1177_13623613241254619 for Associations between autistic and comorbid somatic problems of gastrointestinal disorders, food allergy, pain, and fatigue in adults by Yiran Li, Tian Xie, Harold Snieder and Catharina A Hartman in Autism
