# Supplementary material for: Associations between autistic and comorbid somatic problems of gastrointestinal disorders, food allergy, pain, and fatigue in adults
Source: Autism. 2024 May 30;28(12):3105–17. doi: 10.1177/13623613241254619 (PMC11575103; doi:10.1177/13623613241254619)
Supplement: sj-docx-1-aut-10.1177_13623613241254619 – Supplemental material for Associations between autistic and comorbid somatic problems of gastrointestinal disorders, food allergy, pain, and fatigue in adults [file sj-docx-1-aut-10.1177_13623613241254619.docx]

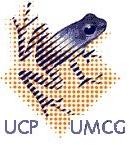


ASBQ self report

Your name:

Your date of birth:

Current date:

On the following pages you will find a list of descriptions of social behavior. Please indicate the extent to which the description applies to you. Please base your answer on your behavior over the **last two months**.

Please mark ‘**clearly applies**’ if the description clearly applies to you.

Please mark ‘**somewhat applies**’ if the description somewhat applies to you. Please mark ‘**does not apply**’ if the description does not apply to you.

Please fill in the questionnaire **as *you* see yourself**, even if this view is not shared by others.

If you make a mistake, please mark the correct answer in black and put a cross through the incorrect answer.


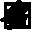

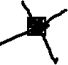

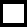


|  |  | **does not apply** | **somewhat applies** | **clearly applies** |
| --- | --- | --- | --- | --- |
| **1.** | You find it difficult to put yourself in someone else’s shoes, for example, you  can’t see why someone is angry. | 🞏 | 🞏 | 🞏 |
| **2.** | You don’t get jokes. | 🞏 | 🞏 | 🞏 |
| **3.** | You panic when things turn out differently than you are used to. | 🞏 | 🞏 | 🞏 |
| **4.** | You feel the urge to flap your hands or arms about when you are excited. | 🞏 | 🞏 | 🞏 |
| **5.** | You take everything literally, for example, you don’t understand certain  expressions. | 🞏 | 🞏 | 🞏 |
| **6.** | You don’t differentiate between friends and strangers, for example, you don’t  care who you are with. | 🞏 | 🞏 | 🞏 |
| **7.** | The reason why you would contact others is to get things done rather than  because you are interested in them. | 🞏 | 🞏 | 🞏 |
| **8.** | You are very naive; you believe everything you are told. | 🞏 | 🞏 | 🞏 |
| **9.** | It takes you ages to get used to somewhere new. | 🞏 | 🞏 | 🞏 |
| **10.** | You resist change; if it were left up to you, everything would stay the same. | 🞏 | 🞏 | 🞏 |
| **11.** | You feel the urge to rock back and forth. | 🞏 | 🞏 | 🞏 |
| **12.** | You feel the urge to make strange, quick movements with your hands or fingers. | 🞏 | 🞏 | 🞏 |
| **13.** | You don’t notice when others make fun of you. | 🞏 | 🞏 | 🞏 |
| **14.** | You want to do certain things in exactly the same way every time. | 🞏 | 🞏 | 🞏 |
| **15.** | You find it hard to follow the gist of a conversation – you miss the point. | 🞏 | 🞏 | 🞏 |
| **16.** | You seek contact with anyone and everyone; you show no reserve. | 🞏 | 🞏 |  |
| **17.** | You do not like surprises, for example, unexpected visitors. | 🞏 | 🞏 | 🞏 |
| **18.** | You really enjoy making certain movements and you want to repeat them. | 🞏 | 🞏 | 🞏 |
| **19.** | You do not take the initiative in contacts with other people. | 🞏 | 🞏 | 🞏 |
| **20.** | You are unaware of other people’s emotional needs, for example, you do not  encourage other people or reassure them. | 🞏 | 🞏 | 🞏 |

|  |  | **does not apply** | **somewhat applies** | **clearly applies** |
| --- | --- | --- | --- | --- |
| **21.** | You touch people when it is not suitable, for example, you hug virtual strangers. | 🞏 | 🞏 | 🞏 |
| **22.** | You don’t like a lot of things happening at once. | 🞏 | 🞏 | 🞏 |
| **23.** | You often want to smell objects. | 🞏 | 🞏 | 🞏 |
| **24.** | You have little or no interest in socializing with others. | 🞏 | 🞏 | 🞏 |
| **25.** | The questions you ask are too personal, or you tell others things that are too  personal. | 🞏 | 🞏 | 🞏 |
| **26.** | You find it hard to sense what someone else will like or think is nice. | 🞏 | 🞏 | 🞏 |
| **27.** | You need an explanation before you understand the meaning behind someone’s  words. | 🞏 | 🞏 | 🞏 |
| **28.** | You behave the same wherever you are ; it makes no difference to you whether  you are at home or somewhere else (visiting others, at work, in the street). | 🞏 | 🞏 | 🞏 |
| **29.** | You give answers that are not relevant - because you haven’t really understood  the question. | 🞏 | 🞏 | 🞏 |
| **30.** | You ignore invitations from others to do something with them. | 🞏 | 🞏 | 🞏 |
| **31.** | You are not really bothered by someone else in pain. | 🞏 | 🞏 | 🞏 |
| **32.** | The only contact you have with others is when you have to buy something or  arrange something, for example with people in a shop or in a government office. | 🞏 | 🞏 | 🞏 |
| **33.** | You are fascinated by certain sounds for example the squeaking of a door, the  humming of a fridge, the rustling of paper. | 🞏 | 🞏 | 🞏 |
| **34.** | You don’t notice when someone is upset or has problems. | 🞏 | 🞏 | 🞏 |
| **35.** | You really need fixed routines and things to be predictable. | 🞏 | 🞏 | 🞏 |
| **36.** | You avoid people who try to make contact with you. | 🞏 | 🞏 | 🞏 |
| **37.** | It’s easy to take advantage of you or get you to do other people’s dirty work. | 🞏 | 🞏 | 🞏 |
| **38.** | You don’t enjoy doing things with other people, for example, doing a chore  together or going somewhere together. | 🞏 | 🞏 | 🞏 |
| **39.** | You feel the urge to often touch things to see what they feel like. | 🞏 | 🞏 | 🞏 |
| **40.** | You ask strangers for things you need, for example for food or drink if you are  hungry or thirsty. | 🞏 | 🞏 | 🞏 |
| **41.** | You hate it when plans are changed at the last moment. | 🞏 | 🞏 | 🞏 |
| **42.** | You really revel in certain colors, shapes or moving objects. | 🞏 | 🞏 | 🞏 |
| **43.** | You are a loner, even in a group you hold yourself apart. | 🞏 | 🞏 | 🞏 |
| **44.** | You don’t show sympathy when others hurt themselves or are unhappy. | 🞏 | 🞏 | 🞏 |

Copyright © C.A. Hartman. UMCG, PO Box 30.001, 9700 RB Groningen. 2013. All rights reserved. No part of this document may be reproduced, stored in a retrieval system, or transmitted in any form or by any means, electronic, mechanical, photocopying, recording or otherwise, without prior permission in writing.
