# Supplementary material for: Associations between autistic and comorbid somatic problems of gastrointestinal disorders, food allergy, pain, and fatigue in adults
Source: Autism. 2024 May 30;28(12):3105–17. doi: 10.1177/13623613241254619 (PMC11575103; doi:10.1177/13623613241254619)
Supplement: sj-docx-2-aut-10.1177_13623613241254619 – Supplemental material for Associations between autistic and comorbid somatic problems of gastrointestinal disorders, food allergy, pain, and fatigue in adults [file sj-docx-2-aut-10.1177_13623613241254619.docx]

Supplementary Materials

Table S1. Associations between probable autism^a^ and somatic problems

| Predictors | Somatic problems | | | |
| --- | --- | --- | --- | --- |
|  | Categorical outcomes | | Continuous outcomes | |
|  | Irritable bowel syndrome (yes/no) | Food allergy  (likely food allergy/indeterminate/no) | Pain  (scale from −0.91 to 6.15) | Fatigue  (scale from −1.39 to 3.75) |
|  | OR | OR | *b* | *b* |
|  | (95% CI) | (95% CI) | (95% CI) | (95% CI) |
| Probable autism^a^ | 4.41^***^ | 1.09 | 0.85^***^ | 1.21^***^ |
|  | (2.65, 7.13) | (0.62, 1.82) | (0.66, 1.05) | (1.01, 1.41) |
|  |  |  |  |  |
| Sex (male)^b^ | 0.45^***^ | 0.50^***^ | −0.30^***^ | −0.12^***^ |
|  | (0.40, 0.52) | (0.46, 0.54) | (−0.33, −0.28) | (−0.15, −0.10) |
|  |  |  |  |  |
| Age | 0.86^***^ | 0.94^***^ | −0.02^***^ | −0.13^***^ |
|  | (0.81, 0.92) | (0.90, 0.98) | (−0.03, −0.01) | (−0.14, −0.11) |
|  |  |  |  |  |
| Age^2^ | 0.99 | 0.94^***^ | 0.04^***^ | −0.02^***^ |
|  | (0.95, 1.04) | (0.91, 0.97) | (0.03, 0.05) | (−0.03, −0.01) |
|  |  |  |  |  |
| Education years | 0.96 | 1.05^*^ | −0.06^***^ | −0.04^***^ |
|  | (0.90, 1.03) | (1.00, 1.10) | (−0.08, −0.05) | (−0.06, −0.03) |
|  |  |  |  |  |
| Employment | 0.99 | 1.02 | −0.01 | −0.01 |
|  | (0.93, 1.06) | (0.97, 1.06) | (−0.02, 0.01) | (−0.02, 0.01) |
|  |  |  |  |  |
| Income | 0.93^*^ | 1.03 | −0.05^***^ | −0.06^***^ |
|  | (0.88, 0.99) | (0.99, 1.07) | (−0.06, −0.04) | (−0.07, −0.04) |
| NSES | 0.94^*^ | 0.98 | −0.01 | −0.02^**^ |
|  | (0.89, 1.00) | (0.94, 1.02) | (−0.02, 0.01) | (−0.03, −0.01) |
|  |  |  |  |  |
| Autism × sex (male)^b^ | 1.26 | 1.24 | −0.33^**^ | −0.15 |
|  | (0.67, 2.33) | (0.64, 2.39) | (−0.56, −0.10) | (−0.38, 0.09) |
|  |  |  |  |  |
| Autism × age | 1.47^*^ | 0.65 | 0.21^***^ | 0.09 |
|  | (1.05, 2.04) | (0.36, 1.02) | (0.09, 0.34) | (−0.03, 0.22) |
|  |  |  |  |  |
| Autism × age^2^ | 0.93 | 0.90 | −0.04 | −0.03 |
|  | (0.72, 1.16) | (0.64, 1.18) | (−0.13, 0.05) | (−0.12, 0.06) |

*Note*. OR, odds ratio; CI: confidence interval; NSES: neighborhood socioeconomic status.

Logistic regression models were used for irritable bowel syndrome and food allergy. Linear regression models were used for pain and fatigue.

^a^probable autism was classified by the following criteria:

First, the total score exceeded the cut-off score according to the prevalence in the general population which was set to 2%. Second, the age of onset was before 18 years old, and that impairment level of at least 5 points on a scale from 1 to 10 was reported. This yielded a prevalence of 1.2% (433/35048, 235 males, 198 females).

^b^Female was the reference category

^*^*p* < .05, ^**^*p* < .01, ^***^*p* < .001

Figure S1. Associations between probable autism^a^ and somatic problems by age


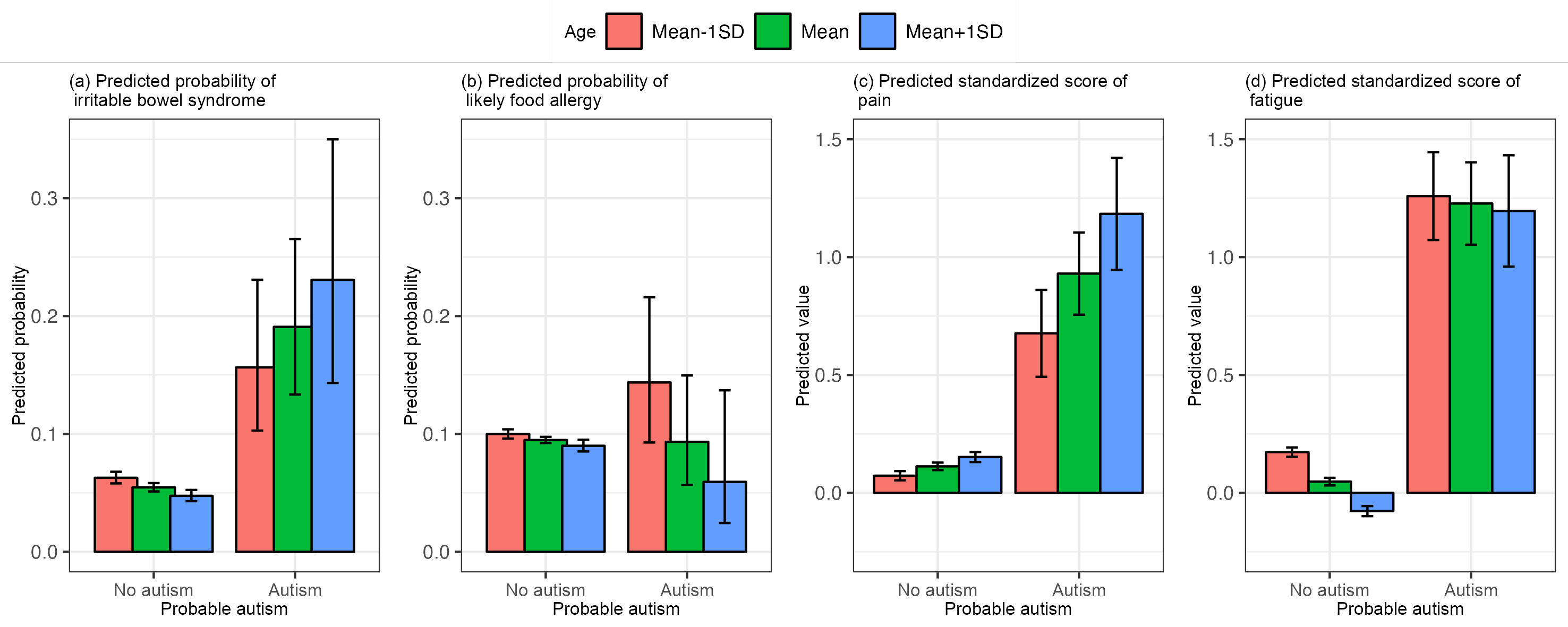


*Note*. *SD*: standard deviation.

Logistic regression models were used for irritable bowel syndrome and likely food allergy. Linear regression models were used for pain and fatigue.

^a^probable autism was classified by the following criteria:

First, the total score exceeded the cut-off score according to the prevalence in the general population which was set to 2%. Second, the age of onset was before 18 years old, and an impairment level of at least 5 points on a scale from 1 to 10 was reported. This yielded a prevalence of 1.2% (433/35048, 235 males, 198 females).

The bars show the means of predicted probabilities or value of outcomes, and the error bars show the 95% confidence intervals of the means.

Figures S1a and S1c show additive and interactive effects of age and probable autism. The associations of probable autism with irritable bowel syndrome and pain were stronger in older adults. Figure S1d shows the additive effects of age and probable autism. Figure S1b shows the additive effect of age.

Figure S2. Associations between probable autism^a^ and somatic problems by sex


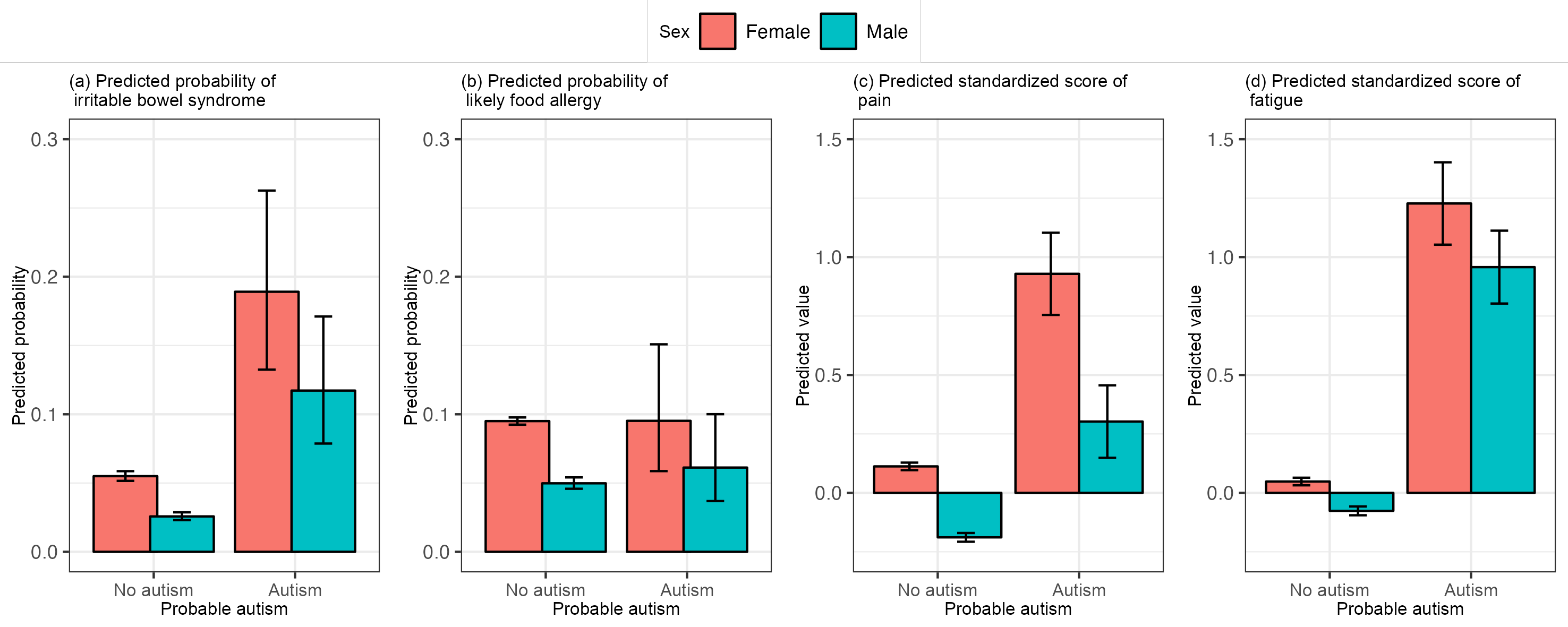


*Note*. Logistic regression models were used for irritable bowel syndrome and likely food allergy. Linear regression models were used for pain and fatigue.

^a^probable autism was classified by the following criteria:

First, the total score exceeded the cut-off score according to the prevalence in the general population which was set to 2%. Second, the age of onset was before 18 years old, and an impairment level of at least 5 points on a scale from 1 to 10 was reported. This yielded a prevalence of 1.2% (433/35048, 235 males, 198 females).

The bars show the means of predicted probabilities or value of outcomes, and the error bars show the 95% confidence intervals of the means.

Figure S2c shows additive and interactive effects of sex and probable autism. The association of autism with pain was stronger in females. Figures S2a and S2d show additive effects of sex and probable autism. Figure S2b shows the additive effect of sex.

Table S2. Associations between scores of autism sub-scales and somatic problems

| Predictors | Somatic problems | | | |
| --- | --- | --- | --- | --- |
|  | Categorical outcomes | | Continuous outcomes | |
|  | Irritable bowel syndrome (yes/no) | Food allergy  (likely food allergy/indeterminate/no) | Pain (scale from −0.91 to 6.15) | Fatigue (scale from −1.39 to 3.75) |
|  | OR | OR | *b* | *b* |
|  | (95% CI) | (95% CI) | (95% CI) | (95% CI) |
| Reduced contact | 1.37^***^ | 1.05 | 0.15^***^ | 0.32^***^ |
|  | (1.28, 1.47) | (0.99, 1.11) | (0.13, 0.17) | (0.30, 0.34) |
| Reduced social insight | 1.31^***^ | 1.10^***^ | 0.15^***^ | 0.28^***^ |
|  | (1.23, 1.40) | (1.04, 1.16) | (0.13, 0.17) | (0.26, 0.30) |
| Reduced empathy | 1.09 | 1.07^*^ | 0.07^***^ | 0.19^***^ |
|  | (0.99, 1.19) | (1.00, 1.15) | (0.05, 0.10) | (0.17, 0.21) |
| Violation of social conventions | 1.22^***^ | 1.09^**^ | 0.12^***^ | 0.18^***^ |
|  | (1.13, 1.32) | (1.03, 1.16) | (0.10, 0.14) | (0.16, 0.20) |
| Difficulty with change | 1.41^***^ | 1.12^***^ | 0.18^***^ | 0.35^***^ |
|  | (1.32, 1.51) | (1.06, 1.18) | (0.16, 0.20) | (0.33, 0.37) |
| Stereotyped sensory−motor behaviors | 1.32^***^ | 1.16^***^ | 0.19^***^ | 0.23^***^ |
|  | (1.24, 1.41) | (1.10, 1.21) | (0.17, 0.21) | (0.22, 0.25) |

*Note*. OR, odds ratio; CI: confidence interval.

Logistic regression models were used for irritable bowel syndrome and likely food allergy. Linear regression models were used for pain and fatigue.

Covariates including sex, age, age^2^, education years, employment, income, neighborhood socio-economic status, autism × sex, autism × age, autism × age^2^ were adjusted.

^*^*p* < .05, ^**^*p* < .01, ^***^*p* < .001
